# Dynamic of Plant Composition and Regeneration following Windthrow in a Temperate Beech Forest

**DOI:** 10.1155/2014/421457

**Published:** 2014-07-21

**Authors:** Sakineh Mollaei Darabi, Yahya Kooch, Seyed Mohsen Hosseini

**Affiliations:** Faculty of Natural Resources & Marine Sciences, Tarbiat Modares University, Mazandaran, Noor 46417-76489, Iran

## Abstract

The effects of soil pedoturbation (*i.e.*, pit and mound microtopography, PM) on development of herbaceous plant species and woody species regeneration were examined in a temperate beech forest (*Fagus orientalis* Lipsky) in northern Iran. We recorded the vegetation in 20 pairs of disturbed and adjacent undisturbed plots and established a chronosequence of PM ages to study the effect of time since microsite formation on cover percent of herbaceous plants and woody regeneration status. According to our findings, *Carex acutiformis* L., *Sambucus ebulus* L., *Brachypodium pinnatum* L., and *Cyclamen coum* L. are found only in the PM microsites, whereas the *Equisetum ramosissimum* L. is recorded only under closed canopy. The coverage percent of *Rubus caesius* L. increased in PM microsites compared to closed canopy intensively. In addition, *Albizia julibrissin* Durazz. is detected in PM microsite, whereas the *Acer cappadocicum * B. and *Prunus persica* L. species were recorded only under closed canopy. We found significant differences in understory species diversity between different ages of PM, and disturbed and adjacent undisturbed plots. Our study supports that the PM complex will create a mosaic of environmental conditions. This environmental heterogeneity could be responsible for the diversity of herbaceous plant species and regeneration of woody species.

## 1. Introduction

In the concept of disturbance ecology, disturbances are no longer understood as the “nemesis of succession” as was wittily commented by Johnson and Miyanishi [1] but are considered as an important part of plant communities dynamics. Individual windthrows (i.e., a pit and related mound formed by the uprooting of a tree, hereafter referred to as PM) create microtopography in the terrain of natural forests [2, 3]. PM usually cover amount of 10 to 35% of the total area in natural forests [4]. In time horizons on the order of 100 to 1000 years, PMs affect every aspect of forest ecosystems [5]. Phenomena occurring on fine spatial scales can therefore function on coarse scales as well, and PMs can affect whole ecosystem dynamics [6]. The extensive literature on the ecological and silvicultural aspects of uprooting and creation of PM is reviewed by Ulanova [7] and Peterson [8]. Ice storms and other factors may cause uprooting, but wind is the most common cause. PM patterns affect the spatial variability of the associations of decomposers, the forest floor, and mineral horizons [9–11], thus affecting herb layer variability and the regeneration of trees [12–14].

Through these processes, PMs influence the formation of subsequent forest generations. Although PMs usually cover a small area in a territory at a given moment, over time horizons, all areas of forest ecosystems can be affected by PMs [5]. In spite of the number of studies that focus on aspects of the PM phenomenon [5, 9–11], as well as more synthetic studies [7], the overall significance of PM disturbances on understory in natural forests has not yet been adequately assessed at different spatial scales. In contrast to the undisturbed forest floor, mounds tend to be exposed to higher light levels; soil temperature is higher, and soil moisture is lower [3]. Increased moisture levels in pits with occasional standing water and thick litter accumulations have often been observed in temperate forest ecosystems [2]. Tree uprooting therefore enhances habitat heterogeneity and provides important microsites for plant establishment. Windthrows create gaps that increase light and free other resources for understory tree and seedling growth [13].

Moreover, it has been demonstrated that the response of understory vegetation is more pronounced in windthrow gaps than in gaps without soil perturbation [15]. Canopy gap creation with soil perturbation can be seen as representing a higher level of disturbance intensity than canopy gap creation without soil perturbation in late successional forest communities [16]. Vascular plant species have differential success on the different types of microsites [17], due to differing physical and chemical processes/properties operating therein [18]. Differences in species composition among the microsites may remain over time as succession occurs on the site as a whole, or the microsites may converge in species composition due to increasing similarity in environmental conditions. There are few long-term studies on PMs beginning immediately after catastrophic windthrow, and processes occurring on recent PMs may be different from those on long-established PMs [19]. In temperature forests, several plant species specialize on particular microsites within the PM complex [20].

The significance of PMs for ecosystem functioning further increases when considering time. In most of studies, the age of windthrows is established indirectly, based on the monitoring of their characteristics in time [21]. Even though the accuracy is low, this method can be applied to a wide range of windthrows and can even be used to assess the role of windthrow dynamics at the level of the whole ecosystem. Data on time sequences of vegetation succession are usually limited to a few years [e.g., 4 to 5 years, 13], while long-term studies not only on tree regeneration but also on herbaceous-layer response are urgently needed [22]. Once created, PMs are not stable habitats but show characteristic changes over time [7]. In a comparative study of mounds with mean ages of approximately 50, 150, and >200 years in coniferous rainforests of southeast Alaska, Den Ouden and Alaback [23] found that the role of windthrow in the maintenance of high understory diversity was more or less restricted to the first decades after the disturbance. With diminishing habitat heterogeneity and environmental conditions that become less favorable for plant establishment, we expected a general decline in species richness on windthrow mounds over time. In Hyrcanian beech (*Fagus orientalis* Lipsky) forests, small-scale disturbances due to canopy gap formation seem to be the prevailing natural disturbance regime [24]. The purpose of this study is to evaluate effects of windthrow events on the understory of beech forests in northern Iran. In particular, we determine whether the plant composition and regeneration of the understory change following disturbance through time and assess the magnitude of the change.

## 2. Materials and Methods

### 2.1. Study Area

**Figure 1:**
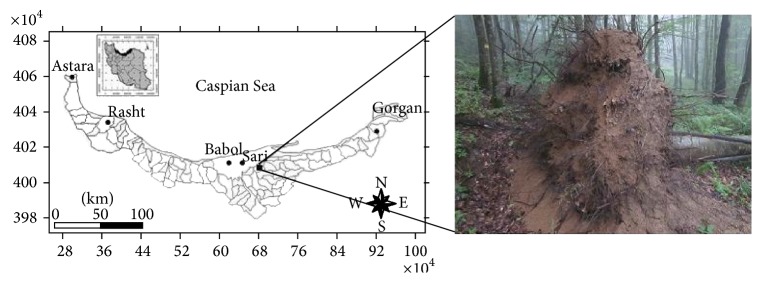
Geographical position of the study site (Mazandaran province, northern Iran) with a photo of uprooted tree.

This research was conducted at the Experimental Forest Station of Darabkola which is located in a temperate forest of Mazandaran province in northern Iran, between 36°35′56′′N and 36°29′23′′N latitudes and 53°18′39′′E and 53°27′20′′E longitudes (Figure 1). This study was carried out in a 34 ha area of the reserve parcel, which is located about 900 m a.s.l. and is covered mostly by* Fagus orientalis* Lipsky (Oriental Beech has an average volume of 187 m^3^ ha^−1^, which represents 74% of total stand volume) mixed with* Carpinus betulus* L. (Common Hornbeam with 21 m^3^ ha^−1^, 8%). Structurally, these forests have high volumes of coarse woody debris with mature forests averaging 51 m^3^ ha^−1^. The parent material is limestone and dolomite limestone belonging to upper Jurassic and lower Cretaceous period. The mean annual temperature, rainfall, and relative humidity are 10°C, 750 mm, and 72.4%, respectively. The easily noticeable uprooted trees offered an ideal opportunity to study and monitor the PMs location, as the fallen trees were not extracted in the whole disturbed area of the forest reserve. Windthrow debris was all left on site on the affected area, a part of which we chose to study was the dynamic of plant composition and regeneration and also natural biogeochemical cycles in such ecosystems. The plots were already incorporated into the net of long-term ecological research and in the experimental platform for ecological research for a systematic assessment and monitoring of the conditions in forest ecosystems after a windthrow.

### 2.2. Data Collection

In the summer of 2013, reserved parcel of Experimental Forest Station of Darabkola was considered. Whole PMs were recorded at the study area. PM ages were considered equal to decay degree of downed tree [25]. Based on examinations dead trees were classified within four groups of decay severity [25] in which decay is recognized visually as follows. Decay degree 1 (young): the tree has recently died and lacks any leaves or blossoms. The bark and tree's appearance have not changed significantly yet (this type of dead tree had died less than 5 years ago). Decay degree 2 (medium): the tree is breaking up; the wood's color has changed to brown and mildew is seen on it but wood is still rough and thick and its chemical and physical characteristics are intact (this type of dead tree has died between 5 and 10 years ago). Decay degree 3 (adult): decay has progressed; the color is changed and chemical and physical features of wood have completely changed. Wood takes a dark color, cracks, and loses its primary form and shape (this type of dead tree has died between 10 and 15 years ago). Decay degree 4 (old): wood has completely spoiled and so called “melt.” It has no resistance against strikes and easily breaks down (this type of dead tree has died more than 15 years ago). In this research, 20 beech uprooted trees (4 decay degrees with 5 replications for each) were selected to study. Circle plots with radius of 4 meters in intersection of PMs were designed for recording cover percent of herbaceous plants and woody regeneration status [24]. Two to three witness trees of the same species were selected in a distance of 20 to 30 m from PM. Percentage of herbaceous plant cover and woody regeneration status were recorded at witness tree locations, under closed canopy position [24].

### 2.3. Diversity Measures

The values of diversity (Simpson index), richness (Margalef index), and evenness (Camargo index) were calculated as follows [26, 27]:

(1) $$\begin{array}{c} 1-D=1-\overset{s}{\underset{i=1}{\sum }}\left[\frac{n_{i(n_{i}-1)}}{N\left(N-1\right)}\right], \end{array}$$

where 1 − *D* is Simpson index; *s* is the number of species; *n*_*i*_ is the number of *i*th species in sample; *N* is the number of all species:

(2) $$\begin{array}{c} R_{1}=\frac{s-1}{L_{n}\left(N\right)}, \end{array}$$

where *R*_1_ is Margalef index; *s* is the number of species; *N* is the number of all species:

(3) $$\begin{array}{c} E=1\cdots 0-\left[\overset{s}{\underset{i=1}{\sum }}\;\overset{s}{\underset{j=1+1}{\sum }}\left[\frac{\left|P_{i}-P_{j}\right|}{s}\right]\right], \end{array}$$

where *E* is Camargo species evenness indexes; *P*_*i*_ is the ratio of *i*th species to all species; *P*_*j*_ is the ratio of *j*th species to all species; *s* is the number of species.

### 2.4. Statistical Analyses

PAST and Ecological methodology software packages were used to calculate species diversity indices. Normality of the variables calculated using the Kolmogrov-Smirnov and Levene tests was used to examine the equality of the variances. Two-way analysis (ANOVA) using GLM procedure was employed for comparison of species diversity index and woody regeneration status in PM different ages and microsites. Whole interactions among treatment were also considered. Duncan's test was used to separate the averages of the dependent variables which were significantly affected by treatment. Significant differences among treatment averages for different parameters were tested at *P* ≤ 0.05. SPSS v. 16 software was used for all the statistical analyses.

## 3. Results

Whole of herbaceous and woody species were identified and recorded in the study area (Table 1). According to our finding, 22 herbaceous and 10 woody species were detected (Table 1). The Cryptophytes and Phanerophytes life forms were dominated in the herbal and regeneration layers, respectively (Table 1). The Hyrcanian chorotype and Gramineae family were the most in the study area (Table 1). Plant coverage and regeneration densities of whole species were imposed by PMs microsites (Table 2). In addition, PM creation and also their different ages influenced presence-absence of some herbaceous and woody species (Table 2). In the herbal layer,* Carex acutiformis* L.,* Sambucus ebulus* L.,* Brachypodium pinnatum* L., and* Cyclamen coum* L. were found only in the PM microsites (Table 2), whereas the* Equisetum ramosissimum* L. was recorded only under closed canopy (Table 2). The coverage percent of* Rubus caesius* L. increased in PM microsites compared to closed canopy intensively (Table 2). In the regeneration layer,* Albizia julibrissin* Durazz. was found in PM microsite, whereas the* Acer cappadocicum* B. and* Prunus persica* L. species were recorded only under closed canopy (Table 2).

Regarding our results, characteristics of plant diversity and regeneration status, thus resulting in PM microsites that may strongly differ with respect to the closed canopy (Table 3). The most various Simpson index among different ages of PM allocate to oldness, adultness, middle-agedness, and youngness (for herbal layer) and oldness, adultness, youngness, and middle-agedness, respectively, (for regeneration layer) in accordance with different plant species (Table 3). PM microsites presented more diversity than closed canopy in both herbal and regeneration layer (Table 3). Plant richness, Margalef index, was increased in older ages of PM in the regeneration layer, whereas the variability was found nonsignificant in the herbal layer (Table 3). Richness never shows any significant difference statistically in accordance with plant species and reproduction aggregation in PM and closed canopy positions (Table 3). Evenness index, Camargo, was significantly greater under closed canopy compared with PM microsites, whereas different ages of PM did not reveal any significant differences (Table 3).

## 4. Discussion

The Hyrcanian vegetation zone, also called Caspian forest, is rich in plant species and covers the southern coasts of the Caspian Sea [28]. According to our findings, having some elements such as cryptophytes and phanerophytes abundantly and in high percentage reveals the characteristics of temperate forests. On the other hand, having fern elements in high percentage in biological forms as, for example, cryptophytes, shows too much humidity in the forest area studied. Moreover lack of some plant elements like Trophytes, which is the index of dried and semidried habitats, is another reason for high humidity percentage in these areas. Investigating the botanical ecological elements in the area shows that Hyrcania chorotype has the most species in population. Noticing this fact, the Experimental Forest Station of Darabkola displays one of the most remarkable characteristics in Hyrcania forests, since having abundant rain, suitable temperature, constant wet which is for being in Caspian Sea vicinity as well the least snowy and icy days, all are the traits of Hyrcania forests [28] which confirm the high amount of Hyrcania elements.

In our research, the presence of PM significantly affects the development of the herb layer as well as the tree regeneration, thus influencing subsequent forest generation. These findings are corroborated by other researchers [13, 14, 29–31]. Some of herbaceous species (i.e.,* Carex acutiformis* L.,* Sambucus ebulus* L.,* Brachypodium pinnatum* L., and* Cyclamen coum* L.) were found only in the PM microsites, whereas the* Equisetum ramosissimum* L. was recorded only under closed canopy. Our results confirm that groups of species differing with respect to important life history traits show different responses to soil disturbance [32, 33]. Previous studies [34–41] have observed an increase in both the frequency and cover of pioneer species (in particular* Rubus*) and ruderal species on disturbed soil. As expected, we found an increase in the disturbance specialists* Rubus caesius* L. in our research.* Rubus* is an opportunistic species that increases in abundance after canopy opening [42, 43] and will decrease with canopy closure [44]. Peterson and Carson [45] suggested that dominance by* Rubus* after disturbances is a function of the presence of propagules. In contrast, the current study suggests that it can be influenced by the “release” of existing plants. The same result is reported by Palmer et al. [34]. The dominant role of* Rubus* in the first decade after windthrow has been reported for many northern temperate, northern hardwoods, and boreal forests [34, 35].

Härdtle et al. [46] showed that the number of ground layer species in undisturbed patches of the forest floor was mainly affected by the canopy closure in acidophytic beech forests of northern Germany. This has been explained by the large proportion of heliophilous species among the acid tolerant species of these forest communities. In our study we suspect that this relationship is also true for disturbed patches of the forest floor. Furthermore, our results suggest that the assemblages of species are a function of decay class. Comparing PM of different age classes revealed that the time since mound formation is a major factor determining plant species richness and composition. This observation is in general agreement with the literature [7, 34]. However, considerable differences have been found in the development of changes in species richness and composition through time and the temporal dimensions of these changes.

It is clear that PMs have profoundly enhanced floristic heterogeneity. The newly exposed soil on the mounds developed a distinctive species composition. Although the “old soil” part of the mound originally had a composition similar to the understory, it developed characteristics of the new soil (both in terms of species composition and growth form distribution) as the surface eroded. The composition and growth form distribution of pits were intermediate between mounds and the understory. The initial differences in species composition among microsites largely disappeared by the last year of the study, and patterns of species richness have mirrored those of the understory quadrates. Since studies of very old PMs [19] find that they possess distinct microcommunities, it will be interesting to see whether this study's PM microtopography will continue to influence species composition in the future. However, response of the herbaceous layer to disturbance is more difficult to predict than tree layer response. The greater variety of species and life history strategies in the herbaceous layer relative to the tree layer suggest that tree-based disturbance-response models [47] are too simple to be applied directly to the herbaceous layer.

On the other hand, PM can cause significant soil perturbation, destroying regeneration by relocation of root systems and through fallen logs [7]. Exposure of bare mineral soil and the creation of microhabitats with varying environmental conditions create various regeneration scenarios [12, 48, 49]. The extent of changes in ground vegetation and properties for regeneration after windthrow depends on disturbance severity, stand composition and structure [8], and soil conditions [2, 3]. PMs produced by the uprooting are important for the recruitment of pioneer trees in mature forests [50]. The response of seedling occurrence to canopy state was different among species. According to our findings,* Albizia julibrissin Durazz. *was detected in PM microsite, whereas the* Acer cappadocicum* B. and* Prunus persica* L. species were recorded only under closed canopy. So, difference of favorable microsites for seedling occurrence would affect the seedling occurrence under gaps. In forests with PM microtopography, it is common to find many trees growing on mounds [7]. Some plants preferentially grow in pit microsites [51]. On mounds, large seeds of species such as* Fagus* are easily visible to rodents and birds or cannot easily lodge in the soil. Other seeds may later be concealed by litter in pits and germinate [52]. As the same, Ilisson et al. [13] pointed that the occurrence of mounds reduced* Fagus sylvatica* establishment. He related lower seedling densities on mounds to soil instability and drought. Kooch et al. [3] claimed that pits seem to be more favourable for the regeneration of water and nutrient-demanding species such as beech, while mounds seem more suitable for the regeneration of more frugal and pioneer species such as hornbeam. We suggest that environmental fluctuations, particularly extreme soil dryness and high surface temperatures [2], prevent the successful establishment of mesophytic species on mounds. While we do not have quantitative soil moisture measurements from both pits and mounds, it seems likely that moisture is a primary factor contributing to differential establishment in these microsites. It is unlikely that the limited amount of soil of mounds, positioned up to 2 m above the surrounding surface, can retain much water. Moreover, in closed forest, Beatty and Stone [53] reported significantly lower soil moisture on older mounds than in pits. The actual physical instability of the mounds probably also prevents some establishment [54]. We have observed young tree seedlings that have fallen from mounds and died when the soil in which they were rooted became detached from the mound.

Our research showed that characteristics of plant diversity and regeneration status, thus resulting in PM microsites that may strongly differ with respect to the closed canopy. Relative to closed canopy, PMs have higher species diversity and greater tree seedling density. The occurrence of these microsites will thus promote increased diversity of the disturbed area as a whole, as compared to areas in which microsite types are fewer or more similar to one another [55]. Many authors have shown that plant diversity associated with microtopographic features is highest in the first years after disturbance [14, 34]. However, considerable differences have been found in the development of changes in species richness and composition through time and the temporal dimensions of these changes are in general agreement with the literature [7, 34, 56].

## 5. Conclusion

Small-scale windthrow gaps with soil pedoturbation (i.e., PM microtopography) provide opportunity for development and dynamic of herbaceous plant species and woody species regeneration in old-growth forests of Iran. Our study supports that the PM complex will create a mosaic of environmental conditions. This environmental heterogeneity could be responsible for the diversity of plant species. Our study supports the view that groups of species differing in important life history traits exhibit different responses to soil disturbance. This should be taken into consideration by forest managers who can prevent or promote tree uprooting by increasing the physical stability of forests with appropriate forest management that attempts to emulate natural processes.

## Figures and Tables

**Table 1:** Floristic list of herbaceous and regeneration species in the study area.

	Species	Life form	Chorotype	Family
Herbal layer	*Carex acutiformis* L.	Cr	H-M	Cyperaceae
	*Rubus caesius* L.	Ph	H	Urticaceae
	*Pteris cretica* L.	Cr	POL	Pteridaceae
	*Asperula odorata* L.	He	H-M	Rubiaceae
	*Conyza bonariensis* L.	He	POL	Compositae
	*Lamium album* L.	Cr	H	Labiatae
	*Oplismenus undulatifolius* P.	Cr	H-M-IT	Gramineae
	*Viola odorata* L.	He	H-M	Violaceae
	*Ruscus hyrcanus* L.	Ph	H	Liliaceae
	*Phyllitis scolopendrium* L.	Cr	H	Asteliaceae
	*Solanum kieseritzk*i L.	Cr	H	Umbelliferae
	*Epimedium pinnatum* L.	He	H	Podophyllaceae
	*Danae racemosa* L.	Ph	H	Liliaceae
	*Hedera pastuchovii* Woron. Ex	Ph	H	Araliaceae
	*Sambucus ebulus* L.	He	POL	Caprifoliaceae
	*Brachypodium pinnatum* L.	He	H-M-IT	Gramineae
	*Hyperium ondrosaemus* L.	Ph	H-M	Hypericaceae
	*Equisetum ramosissimum* L.	Cr	H	Equisetaceae
	*Euphorbia amygdaloides* L.	He	H	Gramineae
	*Pteridium aquilinum* L.	Cr	H-M	Polypodiaceae
	*Cyclamen coum* L.	Cr	H-M-IT	Primulaceae
	*Primula heterochroma* L.	He	H	Primulaceae

Regeneration layer	*Diospyros lotus* L.	Ph	H	Ebenaceae
	*Carpinus betulus* L.	Ph	H	Betulaceae
	*Fagus orientalis* Lipsky.	Ph	H	Fagaceae
	*Acer insigne* B.	Ph	H	Aceraceae
	*Parrotia persica* DC.	Ph	H	Hamamelidaceae
	*Albizia julibrissin* Durazz.	Ph	H	Leguminosae
	*Acer cappadocicum* Boiss.	Ph	H	Aceraceae
	*Ficus carica* L.	Ph	POL	Moraceae
	*Prunus persica* L.	Ph	H	Rosaceae
	*Ulmus glabra* Huds.	Ph	H	Ulmaceae

Life form: Ph: Phanerophyte. Cr: Cryptophyte. He: Hemicriptophyte.

Chorotype: H: Hyrcanian. M: Mediterranean. It: Irano-Turanian. Pol: Poly zonal.

**Table 2:** Mean rate of plant coverage and regeneration density in PM and under closed canopy.

	Species	Young				Medium				Adult				Old				Average			
		P	M	PM	CC	P	M	PM	CC	P	M	PM	CC	P	M	PM	CC	P	M	PM	CC
Herbal layer	*Carex acutiformis *L.	6.3	0	6.3	0	0.6	10.2	10.8	0	0.8	0	0.8	0	0	0	0	0	1.92	2.55	4.47	0
	*Rubus caesius *L.	10.3	8.5	18.8	0	9.5	5	14.5	0	29.6	63.3	92.9	2.3	0.83	1	1.83	2.2	12.55	19.45	32	1.1
	*Pteris cretica *L.	1	1	2	1.3	0	0	0	0	0	0.83	0.83	1	2.1	0	2.1	0	0.25	0.45	0.70	0.57
	*Asperula odorata *L.	0	9.5	9.5	1.6	0	0	0	0	0	0	0	0	0	0	0	0	0	2.37	2.37	0.4
	*Conyza bonariensis *L.	0	2.8	2.8	0	0	0	0	0	0	0	0	0	0	1	1	2.1	0	0.95	0.95	0.52
	*Lamium album *L.	0.83	0.8	1.03	0	0.6	0.8	1.4	2.1	0	0	0	0	0	0	0	0	0.98	0.4	1.38	0.52
	*Oplismenus undulatifolius* P.	1	0.83	1.83	6.3	0.8	0	0.8	0	0.5	0	0.5	0	0	0	0	0	0.57	0.2	0.77	1.57
	*Viola odorata *L.	1.6	0	1.6	5.5	0	0	0	8.6	0.3	0	0.3	2.1	0	8	8	2.1	0.47	2	2.47	4.57
	*Ruscus hyrcanus *L.	0	0	0	0.5	1	0	1	0	9.3	0	9.3	0	0	0	0	7.3	2.57	0	2.57	1.95
	*Phyllitis scolopendrium *L.	6	0	6	6	1	0	1	1	0	0	0	0	0	0	0	0	1.75	0	1.75	1.75
	*Solanum kieseritzki* L.	0.5	2.1	2.6	0.5	0.5	0	0.5	2.1	0	6	6	0	0.5	0	0.5	0	0.37	2.25	2.62	0.65
	*Epimedium pinnatum *L.	0	0	0	0.8	0	0.5	0.5	0	0.6	1	1.6	0	0	0	0	0.6	0.15	0.37	0.52	0.35
	*Danae racemosa *L.	0.9	0.8	1.7	0.3	0	0	0	0	0	0	0	0	0	0	0	0	0.22	0.2	0.42	0.07
	*Hedera pastuchovii *Woron. Ex	0.6	0.5	1.1	0	0	2.3	2.3	0.5	8	0	8	0	0	0	0	0	0.35	0.7	1.05	0.12
	*Sambucus ebulus *L.	2.5	0	2.5	0	0	0	0	0	0	0	0	0	0	0	0	0	0.62	0	0.62	0
	*Brachypodium pinnatum *L.	1.2	0	1.2	0	0	0	0	0	0	0	0	0	0	0	0	0	0.3	0	0.3	0
	*Hyperium ondrosaemus *L.	1.2	0		1.3	0	0	0	0	0	0	0	0.5	0	0	0	0.5	0.3	0	0.3	0.57
	*Equisetum ramosissimum *L.	0	0	0	1.3	0	0	0	0.8	0	0	0	1.3	0	0	0	0	0	0	0	o.85
	*Euphorbia amygdaloides *L.	0.6	0	0.6	1.2	0	0	0	2.1	0	0	0	0	2.1	0.63	2.73	0	0.2	0.57	0.77	0.82
	*Pteridium aquilinum *L.	0	0	0	1.5	0	0	0	0.8	0	1.2	1.2	0.8	0	0	0	0	0	0.85	0.85	0.77
	*Cyclamen coum *L.	0	0	0	0	0	0	0	0	0	0.6	0.6	0	0	0	0	0	0	0.82	0.82	0
	*Primula heterochroma *L.	0	0	0	0	0	0	0	0.6	0	0	0	0.8	0	0	0	0	0	0.77	0.77	0.35

Regeneration layer	*Diospyros lotus *L.	5.6	1.6	7.2	0.8	0.8	0.4	1.2	1.5	0	0	0	0	1.2	0.6	1.26	0.2	1.9	2.15	4.05	2.35
	*Carpinus betulus *L.	4.8	1	5.8	6.6	11	0.6	11.6	6.6	2.6	1.6	4.2	1.2	0.6	1.4	2	7.8	4.75	1.15	5.9	5.55
	*Fagus orientalis *L.	6.6	0.8	7.4	21.6	36.2	3	39.2	24.6	8	3	11	32.6	0.6	4.4	5	12.8	11.95	2.8	14.75	22.9
	*Acer insigne *B.	4.4	1.4	5.8	3.4	4.8	1	5.8	2.8	0.2	0	0.2	0.8	0.6	0	0.6	2	2.5	2.6	5.1	2.85
	*Parrotia persica *DC.	0	0	0	0	0.2	0	0.2	1	1	1	2	2.8	1.6	2.8	4.4	1	0.7	0.95	1.65	1.2
	*Albizia julibrissin Durazz. *	7.6	1.6	8.2	0	0	0	0	0	0	0	0	0	0	0	0	0	1.09	0.4	1.49	0
	*Acer cappadocicum *B.	0	0	0	0.1	0	0	0	0	0	0	0	1	0	0	0	0	0	0	0	0.285
	*Ficus carica *L.	0	0	0	1	0	0	0	1	0	0	0	0	0	0	0	0	0	0.25	0.25	0.25
	*Prunus persica *L.	0	0	0	5	0	0	0	0	0	0	0	0	0	0	0	0	0	0	0	1.25
	*Ulmus glabra *Huds.	0	3	3	2	0	0	0	1	0	0	0	0	0	0	0	0	0	0.75	0.75	0.75

P: pit; M: mound; PM: pit-mound; CC: closed canopy.

**Table 3:** Mean (standard error of mean) plant diversity indices and regeneration status in PM different ages and microsites.

Diversity parameters/treatment	Young		Medium		Adult		Old		Statistic description
	PM	CC	PM	CC	PM	CC	PM	CC	
Simpson (herbal)	0.42 ± 0.05	0.09 ± 0.01	0.56 ± 0.07	0.28 ± 0.09	0.06 ± 0.61	0.09 ± 0.26	0.09 ± 0.60	0.06 ± 0.45	Age: *F* = 2.96, *P* = 0.04 (A4^a^ > A3^ab^, A2^ab^ > A1^b^); Position: *F* = 17.50, *P* = 0.00 (PM^a^ > CC^b^); Age × Position: *F* = 0.50, *P* = 0.68.

Margalef (herbal)	0.84 ± 0.05	0.70 ± 0.05	0.85 ± 0.03	0.36 ± 0.13	0.06 ± 0.83	0.16 ± 0.22	0.08 ± 0.90	0.54 ± 0.02	Age: *F* = 0.24, *P* = 0.86; Position: *F* = 3.35, *P* = 0.07; Age × Position: *F* = 0.21, *P* = 0.88.

Camargo (herbal)	0.32 ± 0.03	0.60 ± 0.04	0.53 ± 0.15	0.71 ± 0.06	0.11 ± 0.38	0.06 ± 0.65	0.10 ± 0.40	0.07 ± 0.68	Age: *F* = 0.78, *P* = 0.50; Position: *F* = 11.40, *P* = 0.00 (CC^a^ > PM^b^); Age × Position: *F* = 0.12, *P* = 0.94.

Simpson (regeneration)	0.01 ± 0.28	0.03 ± 0.39	0.04 ± 0.38	0.05 ± 0.24	0.05 ± 0.54	0.02 ± 0.30	0.02 ± 0.55	0.05 ± 0.35	Age: *F* = 2.91, *P* = 0.04 (A4^a^ > A3^ab^, A1^ab^ > A2^b^); Position: *F* = 9.06, *P* = 0.00 (PM^a^ > CC^b^); Age × Position: *F* = 3.88, *P* = 0.01.

Margalef (regeneration)	0.02 ± 0.61	0.07 ± 0.25	0.01 ± 0.10	0.02 ± 0.12	0.03 ± 0.36	0.03 ± 0.55	0.04 ± 0.46	0.14 ± 0.52	Age: *F* = 3.06, *P* = 0.04 (A4^a^, A3^a^, A1^a^> A2^b^); Position: *F* = 0.04, *P* = 0.84; Age × Position: *F* = 1.33, *P* = 0.27.

Camargo (regeneration)	0.01 ± 0.36	0.04 ± 0.36	0.05 ± 0.28	0.03 ± 0.58	0.07 ± 0.36	0.02 ± 0.40	0.07 ± 0.35	0.04 ± 0.67	Age: *F* = 2.03, *P* = 0.12; Position: *F* = 13.59, *P* = 0.00 (CC^a^ > PM^b^); Age × Position: *F* = 3.39, *P* = 0.03.

*N* = 5 for young, *N* = 5 for medium, *N* = 5 for adult, *N* = 5 for old, *N* = 20 for PM, and *N* = 20 for CC.

Contrasting letters a, b, and c refer to significant differences between ages (young, medium, adult, and old) or pit and mound (PM) and closed canopy (CC) microsites.
